# Supplementary material for: Mixed‐effects models for health care longitudinal data with an informative visiting process: A Monte Carlo simulation study
Source: Stat Neerl. 2019 Sep 5;74(1):5–23. doi: 10.1111/stan.12188 (PMC6919310; doi:10.1111/stan.12188)
Supplement: Supplementary file 1 — STAN_12188‐Supp‐0001‐sim1_suppl.pdf [file STAN-74-5-s001.pdf]

Online Supplementary Material: Mixed effects models for healthcare longitudinal data with an informative visiting process: a Monte Carlo simulation study

Alessandro Gasparini[1], Keith R. Abrams[1], Jessica K. Barrett[2], Rupert W. Major[1,3], Michael J. Sweeting[1,4], Nigel J. Brunskill[3,5], and Michael J. Crowther[1]

[1]Biostatistics Research Group, Department of Health Sciences, University of Leicester, Leicester, United Kingdom
[2]MRC Biostatistics Unit, Cambridge, United Kingdom
[3]Department of Nephrology, University Hospitals of Leicester NHS Trust, Leicester, United Kingdom
[4]Department of Public Health and Primary Care, University of Cambridge, Cambridge, United Kingdom
[5]Department of Infection Immunity and Inflammation, University of Leicester, Leicester, United Kingdom

A Web Appendix 1: Fitting a joint model for the longitudinal and observation process using `merlin`

In this web appendix, we show how to fit a joint model for the longitudinal and observation process using `merlin`. We use one of the simulated datasets as an example:

```
. list id trt adcens obtime t ind y if id <= 5, sepby(id)

     +----------------------------------------------------------------+
     | id   trt     adcens      obtime          t   ind           y |
     |----------------------------------------------------------------|
  1. |  1     1   5.504912   57.912993          0     0   -2.067031 |
     |----------------------------------------------------------------|
  2. |  2     1   8.713062   1.9060644          0     1    .0603457 |
  3. |  2     1   8.713062   1.8949513   1.906064     1    2.305849 |
  4. |  2     1   8.713062   11.255745   3.801016     0    1.666346 |
     |----------------------------------------------------------------|
  5. |  3     1   7.638238   1.4467663          0     1    2.937761 |
  6. |  3     1   7.638238   .64893483   1.446766     1    4.185008 |
  7. |  3     1   7.638238    .8967392   2.095701     1    3.894117 |
  8. |  3     1   7.638238   2.3087203    2.99244     1    3.794495 |
  9. |  3     1   7.638238   1.2613189   5.301161     1    3.807579 |
 10. |  3     1   7.638238   .39773079   6.562479     1    4.377698 |
 11. |  3     1   7.638238    .4048223    6.96021     1    4.841716 |
 12. |  3     1   7.638238   .29073617   7.365033     0    6.266053 |
     |----------------------------------------------------------------|
 13. |  4     1   7.494711   2.3363077          0     1   -1.516442 |
 14. |  4     1   7.494711   11.061691   2.336308     0    .8402521 |
     |----------------------------------------------------------------|
 15. |  5     0   9.304282     1.71188          0     1    .9257486 |
 16. |  5     0   9.304282   3.1208205    1.71188     1    .8777511 |
 17. |  5     0   9.304282   1.2727775     4.8327     1    1.081441 |
 18. |  5     0   9.304282   6.1574917   6.105478     0    2.153026 |
     +----------------------------------------------------------------+
```

In this setting, `id` identifies each individual, `trt` denotes the treatment of interest, `adcens` denotes administrative censoring, `obtime` denotes the time gap between observations, `t` represents the time on a continuous scale, `ind` represents whether there is a next observation or not, and `y` is the longitudinal outcome. The simulated dataset we are using here as an example (`df_4_241.dta`) can be freely downloaded from the public GitHub repository of this manuscript: `https://github.com/ellessenne/infobsmcsim`.

We can first fit a linear mixed model for the longitudinal outcome that disregards the observation process:

```
. mixed y c.t#i.trt || id:

Performing EM optimization:

Performing gradient-based optimization:

Iteration 0:   log likelihood = -1231.3063
Iteration 1:   log likelihood = -1231.3063

Computing standard errors:

Mixed-effects ML regression                     Number of obs     =        706
Group variable: id                              Number of groups  =        200

                                                Obs per group:
                                                              min =          1
                                                              avg =        3.5
                                                              max =         28
```

```
                                                Wald chi2(2)      =     175.01
Log likelihood = -1231.3063                     Prob > chi2       =     0.0000
------------------------------------------------------------------------------
           y |      Coef.   Std. Err.      z    P>|z|     [95% Conf. Interval]
-------------+----------------------------------------------------------------
     trt#c.t |
          0  |   .1645769   .0290451     5.67   0.000     .1076495    .2215043
          1  |   .2362706   .0195091    12.11   0.000     .1980335    .2745078
             |
       _cons |   .5057946   .1387099     3.65   0.000     .2339282    .7776611
------------------------------------------------------------------------------

------------------------------------------------------------------------------
  Random-effects Parameters  |   Estimate   Std. Err.     [95% Conf. Interval]
-----------------------------+------------------------------------------------
id: Identity                 |
                  var(_cons) |   3.120834   .3648008       2.48183    3.924365
-----------------------------+------------------------------------------------
               var(Residual) |   1.035233   .0650994      .9151905    1.171022
------------------------------------------------------------------------------
LR test vs. linear model: chibar2(01) = 552.03        Prob >= chibar2 = 0.0000
```

This helps getting better starting values for the random intercept of the joint model, which we can set manually. This greatly improves convergency rates. The joint model can be easily fitted as follows:

```
. merlin  (y trt t trt#t M1[id] M2[id]@1, family(gaussian)) ///
>         (obtime trt M1[id]@1, family(weibull, failure(ind))) ///
>         , restartvalues(M2 3.120834)

Fitting fixed effects model:

Fitting full model:

Iteration 0:   log likelihood = -2519.2191  (not concave)
Iteration 1:   log likelihood = -2267.8441
Iteration 2:   log likelihood =  -2254.044
Iteration 3:   log likelihood = -2212.1282
Iteration 4:   log likelihood = -2192.0785
Iteration 5:   log likelihood = -2190.4798
Iteration 6:   log likelihood = -2190.4574
Iteration 7:   log likelihood = -2190.4574

Mixed effects regression model                  Number of obs     =        706
Log likelihood = -2190.4574
------------------------------------------------------------------------------
             |      Coef.   Std. Err.      z    P>|z|     [95% Conf. Interval]
-------------+----------------------------------------------------------------
y:           |
         trt |   .8646395   .2767901     3.12   0.002      .322141    1.407138
           t |      .1392   .0287686     4.84   0.000     .0828146    .1955854
       trt#t |   .0728057   .0344989     2.11   0.035     .0051892    .1404223
      M1[id] |   1.280241    .110283    11.61   0.000      1.06409    1.496391
      M2[id] |          1          .        .       .            .           .
       _cons |  -.0258054   .2044809    -0.13   0.900    -.4265807    .3749699
  sd(resid.) |   1.011114   .0313019                      .9515872    1.074364
-------------+----------------------------------------------------------------
obtime:      |
         trt |   1.290468   .2230392     5.79   0.000     .8533188    1.727617
      M1[id] |          1          .        .       .            .           .
       _cons |  -2.852605    .206591   -13.81   0.000    -3.257516   -2.447694
  log(gamma) |  -.0758148    .039647    -1.91   0.056    -.1535215    .0018918
```

```
-------------+----------------------------------------------------------------
id:          |
      sd(M1) |   1.270055   .1068648                      1.076964    1.497767
      sd(M2) |   .6748793   .1077941                      .4934796    .9229602
------------------------------------------------------------------------------
```

As mentioned in the main body of the manuscript, `merlin` supports correlated random effects. We can fit the same model as above assuming correlated random effects by adding the option `covariance(unstructured)`:

```
. merlin  (y trt t trt#t M1[id]@1 M2[id]@1, family(gaussian)) ///
>         (obtime trt M1[id]@1, family(weibull, failure(ind))) ///
>         , restartvalues(M2 3.120834) covariance(unstructured)

Fitting fixed effects model:

Fitting full model:

Iteration 0:   log likelihood = -2495.3693  (not concave)
Iteration 1:   log likelihood = -2237.1071
Iteration 2:   log likelihood = -2194.5249
Iteration 3:   log likelihood =  -2190.514
Iteration 4:   log likelihood = -2190.4574
Iteration 5:   log likelihood = -2190.4574

Mixed effects regression model                  Number of obs     =        706
Log likelihood = -2190.4574
------------------------------------------------------------------------------
             |      Coef.   Std. Err.      z    P>|z|     [95% Conf. Interval]
-------------+----------------------------------------------------------------
y:           |
         trt |   .8646393   .2767899     3.12   0.002      .322141    1.407138
           t |      .1392   .0287686     4.84   0.000     .0828146    .1955854
       trt#t |   .0728057   .0344989     2.11   0.035     .0051891    .1404223
      M1[id] |          1          .        .       .            .           .
      M2[id] |          1          .        .       .            .           .
       _cons |  -.0258062   .2044808    -0.13   0.900    -.4265813    .3749688
  sd(resid.) |   1.011113   .0313019                      .9515872    1.074363
-------------+----------------------------------------------------------------
obtime:      |
         trt |   1.290467    .223039     5.79   0.000     .8533187    1.727615
      M1[id] |          1          .        .       .            .           .
       _cons |  -2.852605   .2065908   -13.81   0.000    -3.257515   -2.447694
  log(gamma) |  -.0758152    .039647    -1.91   0.056    -.1535218    .0018915
-------------+----------------------------------------------------------------
id:          |
      sd(M1) |   1.270054   .1068647                      1.076962    1.497765
      sd(M2) |   .7629827    .085194                      .6130138    .9496404
 corr(M2,M1) |   .4664875   .1614041                      .1008934    .7210867
------------------------------------------------------------------------------
```

Note that in this setting we also constrain the association value to 1: `M1[id]@1`.

`merlin` is available from the Boston College Statistical Software Components (SSC) archive. If not available on a system, it can be installed directly from the Stata console by typing `ssc install merlin`.

B Web Appendix 2: Additional results from Monte Carlo simulation study

Results for all simulated scenarios are tabulated here in Tables B.1 to B.10; values in red are summary statistics that are statistically significantly different than their target value (0 for bias, 95% for coverage), using Z-tests and estimated Monte Carlo standard errors.

Table B.1: Results for DGM: Γ distribution not depending on treatment

	Estimate	SE	Empirical SE	Bias	Coverage	MSE
**Model A: JM**						
α_0	-0.0036	0.0949	0.0931 (0.0021)	-0.0036 (0.0029)	0.9480 (0.0070)	0.0087 (0.0004)
α_1	1.0022	0.1215	0.1195 (0.0027)	0.0022 (0.0038)	0.9560 (0.0065)	0.0143 (0.0006)
α_2	0.2000	0.0131	0.0130 (0.0003)	-0.0000 (0.0004)	0.9530 (0.0067)	0.0002 (0.0000)
**Model B: ME (Total)**						
α_0	-0.0035	0.0947	0.0934 (0.0021)	-0.0035 (0.0030)	0.9470 (0.0071)	0.0087 (0.0004)
α_1	1.0023	0.1214	0.1200 (0.0027)	0.0023 (0.0038)	0.9530 (0.0067)	0.0144 (0.0006)
α_2	0.2000	0.0131	0.0131 (0.0003)	-0.0000 (0.0004)	0.9510 (0.0068)	0.0002 (0.0000)
**Model C: ME (Cumulative)**						
α_0	-0.0069	0.1024	0.0998 (0.0022)	-0.0069 (0.0032)	0.9500 (0.0069)	0.0100 (0.0005)
α_1	1.0023	0.1215	0.1197 (0.0027)	0.0023 (0.0038)	0.9530 (0.0067)	0.0143 (0.0006)
α_2	0.1980	0.0262	0.0269 (0.0006)	-0.0020 (0.0009)	0.9410 (0.0075)	0.0007 (0.0000)
**Model D: ME (No adjustment)**						
α_0	-0.0034	0.0947	0.0931 (0.0021)	-0.0034 (0.0029)	0.9480 (0.0070)	0.0087 (0.0004)
α_1	1.0023	0.1215	0.1194 (0.0027)	0.0023 (0.0038)	0.9560 (0.0065)	0.0143 (0.0006)
α_2	0.2000	0.0131	0.0130 (0.0003)	0.0000 (0.0004)	0.9520 (0.0068)	0.0002 (0.0000)
**Model E: GEE (IIVW)**						
α_0	-0.0030	0.0985	0.0970 (0.0022)	-0.0030 (0.0031)	0.9580 (0.0063)	0.0094 (0.0004)
α_1	1.0026	0.1267	0.1248 (0.0028)	0.0026 (0.0039)	0.9570 (0.0064)	0.0156 (0.0007)
α_2	0.2000	0.0145	0.0145 (0.0003)	0.0000 (0.0005)	0.9500 (0.0069)	0.0002 (0.0000)

Table B.2: Results for DGM: JM ($\gamma = 0.00, \lambda = 0.10$)

	Estimate	SE	Empirical SE	Bias	Coverage	MSE
**Model A: JM**						
α_0	-0.0037	0.1066	0.1027 (0.0023)	-0.0037 (0.0032)	0.9540 (0.0066)	0.0106 (0.0005)
α_1	1.0058	0.1379	0.1357 (0.0030)	0.0058 (0.0043)	0.9590 (0.0063)	0.0184 (0.0008)
α_2	0.1998	0.0165	0.0163 (0.0004)	-0.0002 (0.0005)	0.9500 (0.0069)	0.0003 (0.0000)
**Model B: ME (Total)**						
α_0	-0.0046	0.1065	0.1033 (0.0023)	-0.0046 (0.0033)	0.9580 (0.0063)	0.0107 (0.0005)
α_1	1.0070	0.1437	0.1432 (0.0032)	0.0070 (0.0045)	0.9500 (0.0069)	0.0205 (0.0009)
α_2	0.1997	0.0163	0.0160 (0.0004)	-0.0003 (0.0005)	0.9490 (0.0070)	0.0003 (0.0000)
**Model C: ME (Cumulative)**						
α_0	-0.0047	0.1065	0.1023 (0.0023)	-0.0047 (0.0032)	0.9570 (0.0064)	0.0105 (0.0005)
α_1	1.0056	0.1381	0.1366 (0.0031)	0.0056 (0.0043)	0.9530 (0.0067)	0.0187 (0.0008)
α_2	0.1992	0.0224	0.0223 (0.0005)	-0.0008 (0.0007)	0.9520 (0.0068)	0.0005 (0.0000)
**Model D: ME (No adjustment)**						
α_0	-0.0042	0.1058	0.1020 (0.0023)	-0.0042 (0.0032)	0.9570 (0.0064)	0.0104 (0.0005)
α_1	1.0058	0.1377	0.1359 (0.0030)	0.0058 (0.0043)	0.9570 (0.0064)	0.0185 (0.0008)
α_2	0.1996	0.0160	0.0158 (0.0004)	-0.0004 (0.0005)	0.9470 (0.0071)	0.0003 (0.0000)
**Model E: GEE (IIVW)**						
α_0	-0.0062	0.1160	0.1150 (0.0026)	-0.0062 (0.0036)	0.9390 (0.0076)	0.0132 (0.0006)
α_1	1.0084	0.1575	0.1598 (0.0036)	0.0084 (0.0051)	0.9470 (0.0071)	0.0256 (0.0011)
α_2	0.1993	0.0192	0.0196 (0.0004)	-0.0007 (0.0006)	0.9390 (0.0076)	0.0004 (0.0000)

Plots with bias, coverage, and MSE of the fixed intercept α_0 and the effect of time α_2 are included as Figures B.1 and B.2, respectively.

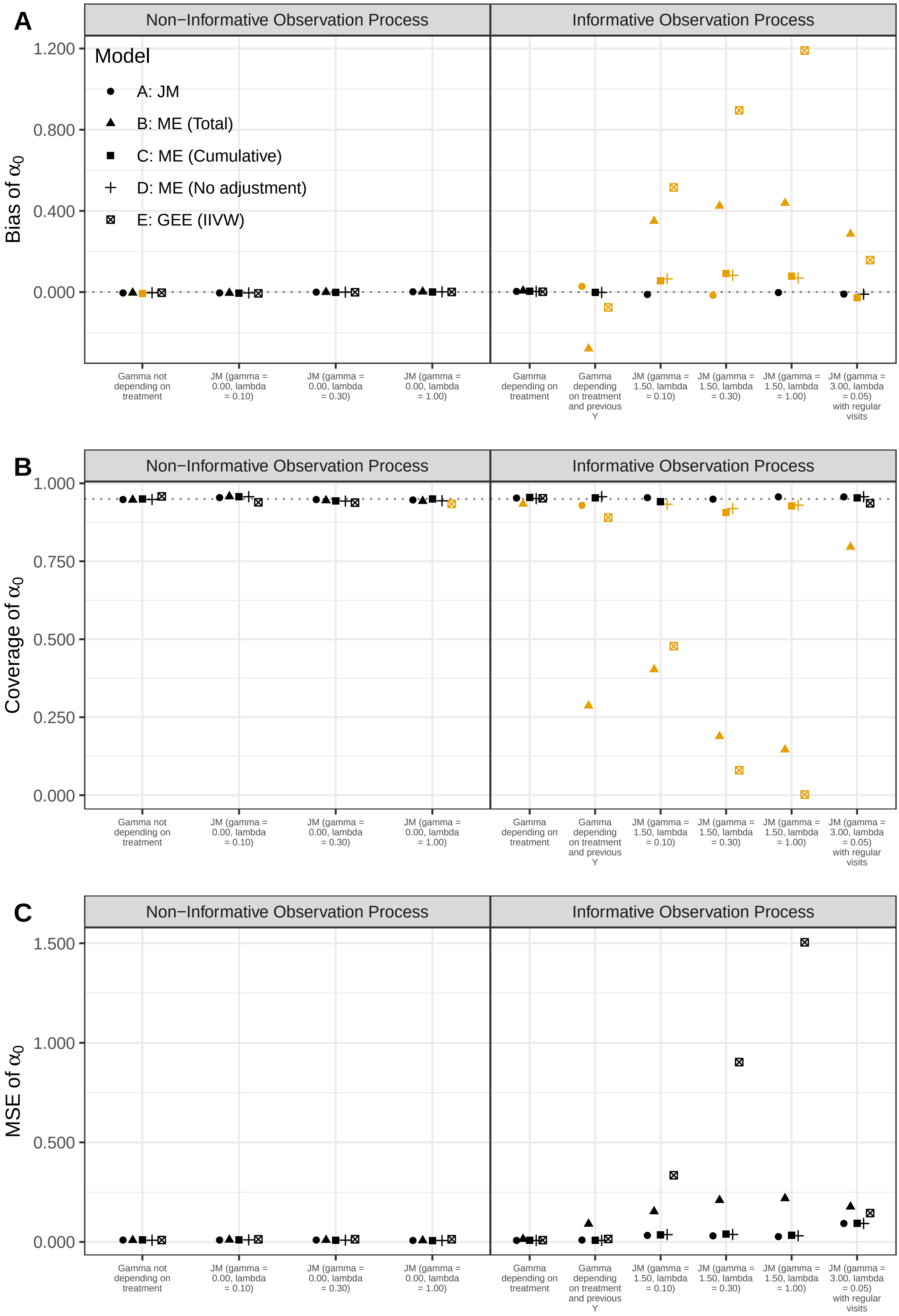

Figure B.1: Bias, coverage, and mean squared error of the intercept α_0. Panel A depicts bias, panel B depicts coverage, and panel C depicts MSE. Orange colour identifies scenarios where the summary statistic was statistically significantly different than the target value (0 for bias, 95% for coverage), using Z-tests and estimated Monte Carlo standard errors.

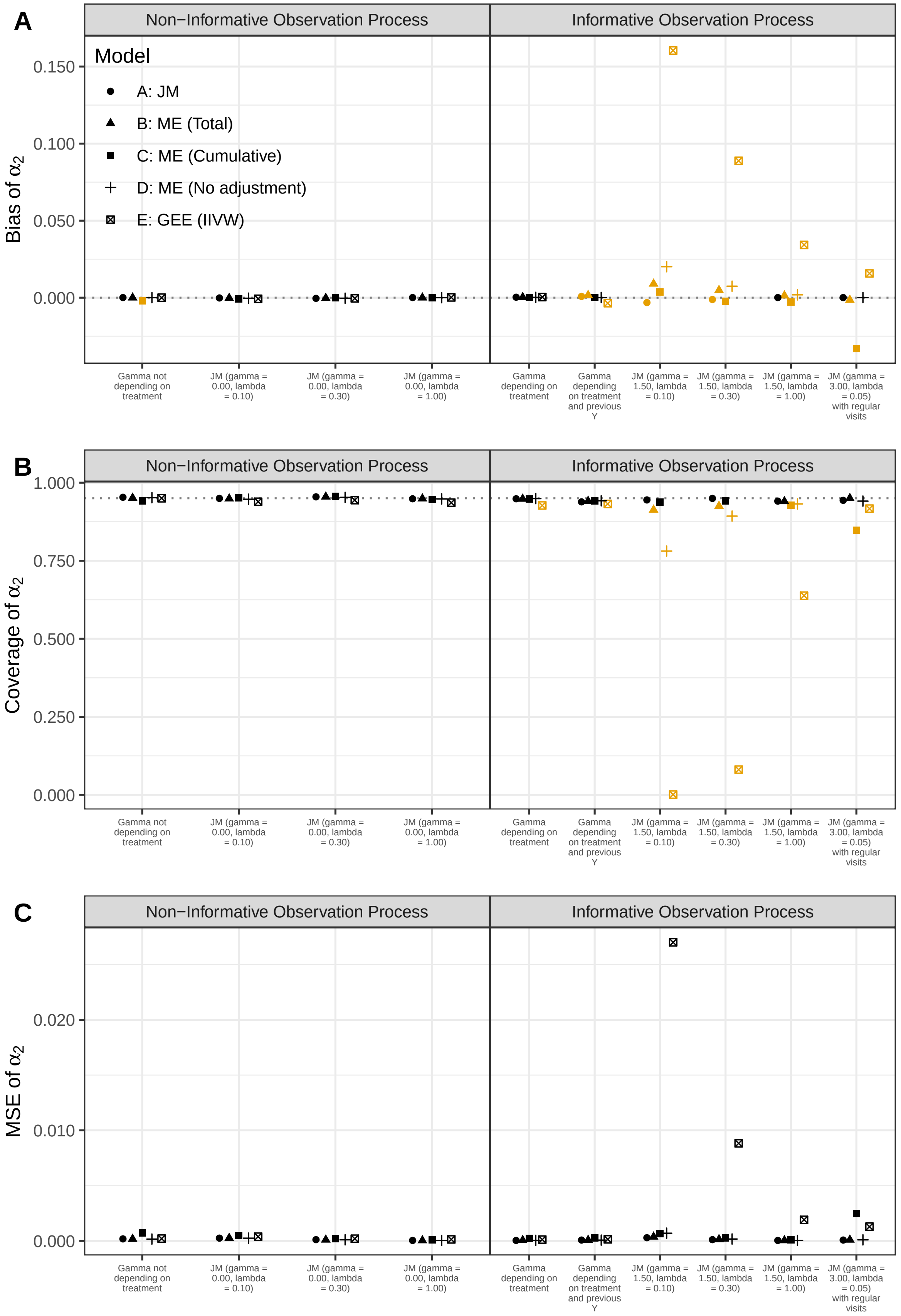

Figure B.2: Bias, coverage, and mean squared error of the effect of time α_2. Panel A depicts bias, panel B depicts coverage, and panel C depicts MSE. Orange colour identifies scenarios where the summary statistic was statistically significantly different than the target value (0 for bias, 95% for coverage), using Z-tests and estimated Monte Carlo standard errors.

Table B.3: Results for DGM: JM ($\gamma = 0.00, \lambda = 0.30$)

	Estimate	SE	Empirical SE	Bias	Coverage	MSE
**Model A: JM**						
α_0	-0.0002	0.0959	0.0961 (0.0021)	-0.0002 (0.0030)	0.9480 (0.0070)	0.0092 (0.0004)
α_1	0.9992	0.1224	0.1257 (0.0028)	-0.0008 (0.0040)	0.9400 (0.0075)	0.0158 (0.0007)
α_2	0.1997	0.0107	0.0102 (0.0002)	-0.0003 (0.0003)	0.9540 (0.0066)	0.0001 (0.0000)
**Model B: ME (Total)**						
α_0	-0.0003	0.0957	0.0971 (0.0022)	-0.0003 (0.0031)	0.9450 (0.0072)	0.0094 (0.0004)
α_1	0.9998	0.1281	0.1322 (0.0030)	-0.0002 (0.0042)	0.9380 (0.0076)	0.0175 (0.0008)
α_2	0.1997	0.0106	0.0102 (0.0002)	-0.0003 (0.0003)	0.9550 (0.0066)	0.0001 (0.0000)
**Model C: ME (Cumulative)**						
α_0	-0.0006	0.0955	0.0966 (0.0022)	-0.0006 (0.0031)	0.9430 (0.0073)	0.0093 (0.0004)
α_1	0.9993	0.1225	0.1263 (0.0028)	-0.0007 (0.0040)	0.9430 (0.0073)	0.0159 (0.0007)
α_2	0.1999	0.0147	0.0143 (0.0003)	-0.0001 (0.0005)	0.9560 (0.0065)	0.0002 (0.0000)
**Model D: ME (No adjustment)**						
α_0	-0.0001	0.0949	0.0955 (0.0021)	-0.0001 (0.0030)	0.9430 (0.0073)	0.0091 (0.0004)
α_1	0.9991	0.1221	0.1255 (0.0028)	-0.0009 (0.0040)	0.9440 (0.0073)	0.0157 (0.0007)
α_2	0.1997	0.0106	0.0101 (0.0002)	-0.0003 (0.0003)	0.9530 (0.0067)	0.0001 (0.0000)
**Model E: GEE (IIVW)**						
α_0	-0.0008	0.1136	0.1172 (0.0026)	-0.0008 (0.0037)	0.9380 (0.0076)	0.0137 (0.0006)
α_1	1.0006	0.1541	0.1587 (0.0035)	0.0006 (0.0050)	0.9530 (0.0067)	0.0252 (0.0012)
α_2	0.1995	0.0143	0.0143 (0.0003)	-0.0005 (0.0005)	0.9440 (0.0073)	0.0002 (0.0000)

Table B.4: Results for DGM: JM ($\gamma = 0.00, \lambda = 1.00$)

	Estimate	SE	Empirical SE	Bias	Coverage	MSE
**Model A: JM**						
α_0	0.0011	0.0837	0.0865 (0.0019)	0.0011 (0.0027)	0.9470 (0.0071)	0.0075 (0.0004)
α_1	0.9977	0.1102	0.1134 (0.0025)	-0.0023 (0.0036)	0.9530 (0.0067)	0.0129 (0.0006)
α_2	0.2000	0.0064	0.0064 (0.0001)	-0.0000 (0.0002)	0.9490 (0.0070)	0.0000 (0.0000)
**Model B: ME (Total)**						
α_0	0.0021	0.0843	0.0866 (0.0019)	0.0021 (0.0027)	0.9430 (0.0073)	0.0075 (0.0004)
α_1	0.9957	0.1158	0.1181 (0.0026)	-0.0043 (0.0037)	0.9510 (0.0068)	0.0139 (0.0006)
α_2	0.2000	0.0064	0.0064 (0.0001)	-0.0000 (0.0002)	0.9490 (0.0070)	0.0000 (0.0000)
**Model C: ME (Cumulative)**						
α_0	0.0014	0.0842	0.0866 (0.0019)	0.0014 (0.0027)	0.9500 (0.0069)	0.0075 (0.0003)
α_1	0.9975	0.1103	0.1135 (0.0025)	-0.0025 (0.0036)	0.9530 (0.0067)	0.0129 (0.0005)
α_2	0.1999	0.0092	0.0090 (0.0002)	-0.0001 (0.0003)	0.9470 (0.0071)	0.0001 (0.0000)
**Model D: ME (No adjustment)**						
α_0	0.0013	0.0831	0.0858 (0.0019)	0.0013 (0.0027)	0.9440 (0.0073)	0.0074 (0.0003)
α_1	0.9976	0.1100	0.1134 (0.0025)	-0.0024 (0.0036)	0.9510 (0.0068)	0.0129 (0.0006)
α_2	0.2000	0.0064	0.0064 (0.0001)	-0.0000 (0.0002)	0.9480 (0.0070)	0.0000 (0.0000)
**Model E: GEE (IIVW)**						
α_0	0.0006	0.1112	0.1163 (0.0026)	0.0006 (0.0037)	0.9340 (0.0079)	0.0135 (0.0007)
α_1	1.0022	0.1519	0.1681 (0.0038)	0.0022 (0.0053)	0.9140 (0.0089)	0.0282 (0.0013)
α_2	0.2002	0.0112	0.0117 (0.0003)	0.0002 (0.0004)	0.9360 (0.0077)	0.0001 (0.0000)

Table B.5: Results for DGM: Γ distribution depending on treatment

	Estimate	SE	Empirical SE	Bias	Coverage	MSE
**Model A: JM**						
α_0	0.0033	0.0890	0.0877 (0.0020)	0.0033 (0.0028)	0.9530 (0.0067)	0.0077 (0.0003)
α_1	0.9952	0.1131	0.1137 (0.0025)	-0.0048 (0.0036)	0.9560 (0.0065)	0.0129 (0.0006)
α_2	0.2002	0.0073	0.0075 (0.0002)	0.0002 (0.0002)	0.9490 (0.0070)	0.0001 (0.0000)
**Model B: ME (Total)**						
α_0	0.0065	0.1177	0.1201 (0.0027)	0.0065 (0.0038)	0.9340 (0.0079)	0.0145 (0.0006)
α_1	0.9884	0.1932	0.1982 (0.0044)	-0.0116 (0.0063)	0.9380 (0.0076)	0.0394 (0.0018)
α_2	0.2002	0.0073	0.0075 (0.0002)	0.0002 (0.0002)	0.9480 (0.0070)	0.0001 (0.0000)
**Model C: ME (Cumulative)**						
α_0	0.0035	0.0940	0.0928 (0.0021)	0.0035 (0.0029)	0.9550 (0.0066)	0.0086 (0.0004)
α_1	0.9949	0.1191	0.1197 (0.0027)	-0.0051 (0.0038)	0.9550 (0.0066)	0.0143 (0.0006)
α_2	0.2001	0.0154	0.0155 (0.0003)	0.0001 (0.0005)	0.9480 (0.0070)	0.0002 (0.0000)
**Model D: ME (No adjustment)**						
α_0	0.0033	0.0888	0.0876 (0.0020)	0.0033 (0.0028)	0.9510 (0.0068)	0.0077 (0.0003)
α_1	0.9952	0.1129	0.1135 (0.0025)	-0.0048 (0.0036)	0.9550 (0.0066)	0.0129 (0.0006)
α_2	0.2002	0.0073	0.0075 (0.0002)	0.0002 (0.0002)	0.9490 (0.0070)	0.0001 (0.0000)
**Model E: GEE (IIVW)**						
α_0	0.0018	0.0965	0.0947 (0.0021)	0.0018 (0.0030)	0.9520 (0.0068)	0.0090 (0.0004)
α_1	0.9975	0.1197	0.1191 (0.0027)	-0.0025 (0.0038)	0.9530 (0.0067)	0.0142 (0.0006)
α_2	0.2004	0.0105	0.0111 (0.0002)	0.0004 (0.0003)	0.9270 (0.0082)	0.0001 (0.0000)

Table B.6: Results for DGM: Γ distribution depending on treatment and previous Y

	Estimate	SE	Empirical SE	Bias	Coverage	MSE
**Model A: JM**						
α_0	0.0279	0.0911	0.0889 (0.0020)	0.0279 (0.0028)	0.9300 (0.0081)	0.0087 (0.0004)
α_1	0.9803	0.1154	0.1114 (0.0025)	-0.0197 (0.0035)	0.9520 (0.0068)	0.0128 (0.0006)
α_2	0.2009	0.0085	0.0085 (0.0002)	0.0009 (0.0003)	0.9380 (0.0076)	0.0001 (0.0000)
**Model B: ME (Total)**						
α_0	-0.2787	0.1095	0.1138 (0.0025)	-0.2787 (0.0036)	0.2870 (0.0143)	0.0906 (0.0021)
α_1	1.5655	0.1745	0.1776 (0.0040)	0.5655 (0.0056)	0.1050 (0.0097)	0.3513 (0.0064)
α_2	0.2016	0.0085	0.0086 (0.0002)	0.0016 (0.0003)	0.9410 (0.0075)	0.0001 (0.0000)
**Model C: ME (Cumulative)**						
α_0	-0.0018	0.0940	0.0924 (0.0021)	-0.0018 (0.0029)	0.9540 (0.0066)	0.0085 (0.0004)
α_1	1.0015	0.1208	0.1166 (0.0026)	0.0015 (0.0037)	0.9550 (0.0066)	0.0136 (0.0006)
α_2	0.2002	0.0167	0.0170 (0.0004)	0.0002 (0.0005)	0.9420 (0.0074)	0.0003 (0.0000)
**Model D: ME (No adjustment)**						
α_0	-0.0017	0.0905	0.0884 (0.0020)	-0.0017 (0.0028)	0.9570 (0.0064)	0.0078 (0.0003)
α_1	1.0016	0.1146	0.1111 (0.0025)	0.0016 (0.0035)	0.9490 (0.0070)	0.0123 (0.0006)
α_2	0.2001	0.0085	0.0085 (0.0002)	0.0001 (0.0003)	0.9420 (0.0074)	0.0001 (0.0000)
**Model E: GEE (IIVW)**						
α_0	-0.0754	0.0979	0.0967 (0.0022)	-0.0754 (0.0031)	0.8900 (0.0099)	0.0150 (0.0006)
α_1	0.9937	0.1226	0.1201 (0.0027)	-0.0063 (0.0038)	0.9420 (0.0074)	0.0145 (0.0007)
α_2	0.1964	0.0112	0.0114 (0.0003)	-0.0036 (0.0004)	0.9320 (0.0080)	0.0001 (0.0000)

Table B.7: Results for DGM: JM ($\gamma = 1.50, \lambda = 0.10$)

	Estimate	SE	Empirical SE	Bias	Coverage	MSE
**Model A: JM**						
α_0	-0.0110	0.1864	0.1805 (0.0041)	-0.0110 (0.0058)	0.9539 (0.0068)	0.0327 (0.0016)
α_1	1.0150	0.2570	0.2514 (0.0058)	0.0150 (0.0081)	0.9466 (0.0073)	0.0633 (0.0030)
α_2	0.1967	0.0166	0.0166 (0.0004)	-0.0033 (0.0005)	0.9445 (0.0074)	0.0003 (0.0000)
**Model B: ME (Total)**						
α_0	0.3499	0.1558	0.1756 (0.0039)	0.3499 (0.0056)	0.4030 (0.0155)	0.1532 (0.0041)
α_1	0.3690	0.2193	0.2362 (0.0053)	-0.6310 (0.0075)	0.1860 (0.0123)	0.4539 (0.0096)
α_2	0.2090	0.0168	0.0171 (0.0004)	0.0090 (0.0005)	0.9130 (0.0089)	0.0004 (0.0000)
**Model C: ME (Cumulative)**						
α_0	0.0547	0.1835	0.1814 (0.0041)	0.0547 (0.0057)	0.9410 (0.0075)	0.0359 (0.0016)
α_1	0.9777	0.2528	0.2501 (0.0056)	-0.0223 (0.0079)	0.9470 (0.0071)	0.0630 (0.0028)
α_2	0.2036	0.0243	0.0254 (0.0006)	0.0036 (0.0008)	0.9380 (0.0076)	0.0007 (0.0000)
**Model D: ME (No adjustment)**						
α_0	0.0649	0.1839	0.1809 (0.0040)	0.0649 (0.0057)	0.9330 (0.0079)	0.0369 (0.0017)
α_1	0.9857	0.2540	0.2499 (0.0056)	-0.0143 (0.0079)	0.9490 (0.0070)	0.0626 (0.0028)
α_2	0.2201	0.0168	0.0172 (0.0004)	0.0201 (0.0005)	0.7810 (0.0131)	0.0007 (0.0000)
**Model E: GEE (IIVW)**						
α_0	0.5156	0.2686	0.2629 (0.0059)	0.5156 (0.0083)	0.4780 (0.0158)	0.3349 (0.0105)
α_1	1.0567	0.3938	0.3939 (0.0088)	0.0567 (0.0125)	0.9530 (0.0067)	0.1582 (0.0082)
α_2	0.3604	0.0339	0.0357 (0.0008)	0.1604 (0.0011)	0.0010 (0.0010)	0.0270 (0.0004)

Table B.8: Results for DGM: JM ($\gamma = 1.50, \lambda = 0.30$)

	Estimate	SE	Empirical SE	Bias	Coverage	MSE
**Model A: JM**						
α_0	-0.0151	0.1793	0.1772 (0.0040)	-0.0151 (0.0056)	0.9490 (0.0070)	0.0316 (0.0014)
α_1	1.0213	0.2478	0.2537 (0.0057)	0.0213 (0.0080)	0.9390 (0.0076)	0.0648 (0.0029)
α_2	0.1987	0.0107	0.0108 (0.0002)	-0.0013 (0.0003)	0.9500 (0.0069)	0.0001 (0.0000)
**Model B: ME (Total)**						
α_0	0.4246	0.1420	0.1719 (0.0038)	0.4246 (0.0054)	0.1890 (0.0124)	0.2098 (0.0049)
α_1	0.2665	0.2001	0.2442 (0.0055)	-0.7335 (0.0077)	0.1010 (0.0095)	0.5976 (0.0112)
α_2	0.2048	0.0108	0.0108 (0.0002)	0.0048 (0.0003)	0.9250 (0.0083)	0.0001 (0.0000)
**Model C: ME (Cumulative)**						
α_0	0.0924	0.1754	0.1768 (0.0040)	0.0924 (0.0056)	0.9070 (0.0092)	0.0397 (0.0018)
α_1	0.9663	0.2421	0.2503 (0.0056)	-0.0337 (0.0079)	0.9390 (0.0076)	0.0637 (0.0028)
α_2	0.1976	0.0155	0.0159 (0.0004)	-0.0024 (0.0005)	0.9410 (0.0075)	0.0003 (0.0000)
**Model D: ME (No adjustment)**						
α_0	0.0824	0.1757	0.1755 (0.0039)	0.0824 (0.0055)	0.9190 (0.0086)	0.0375 (0.0017)
α_1	0.9737	0.2430	0.2495 (0.0056)	-0.0263 (0.0079)	0.9400 (0.0075)	0.0629 (0.0028)
α_2	0.2075	0.0108	0.0109 (0.0002)	0.0075 (0.0003)	0.8930 (0.0098)	0.0002 (0.0000)
**Model E: GEE (IIVW)**						
α_0	0.8957	0.2980	0.3178 (0.0071)	0.8957 (0.0100)	0.0800 (0.0086)	0.9031 (0.0216)
α_1	1.0472	0.4236	0.4561 (0.0102)	0.0472 (0.0144)	0.9340 (0.0079)	0.2100 (0.0111)
α_2	0.2889	0.0271	0.0305 (0.0007)	0.0889 (0.0010)	0.0810 (0.0086)	0.0088 (0.0002)

Table B.9: Results for DGM: JM ($\gamma = 1.50, \lambda = 1.00$)

	Estimate	SE	Empirical SE	Bias	Coverage	MSE
**Model A: JM**						
α_0	-0.0019	0.1717	0.1651 (0.0037)	-0.0019 (0.0052)	0.9560 (0.0065)	0.0272 (0.0012)
α_1	1.0076	0.2393	0.2385 (0.0053)	0.0076 (0.0075)	0.9510 (0.0068)	0.0569 (0.0028)
α_2	0.2001	0.0064	0.0065 (0.0001)	0.0001 (0.0002)	0.9410 (0.0075)	0.0000 (0.0000)
**Model B: ME (Total)**						
α_0	0.4389	0.1314	0.1621 (0.0036)	0.4389 (0.0051)	0.1460 (0.0112)	0.2189 (0.0047)
α_1	0.2312	0.1869	0.2352 (0.0053)	-0.7688 (0.0074)	0.0560 (0.0073)	0.6463 (0.0113)
α_2	0.2013	0.0064	0.0065 (0.0001)	0.0013 (0.0002)	0.9400 (0.0075)	0.0000 (0.0000)
**Model C: ME (Cumulative)**						
α_0	0.0791	0.1687	0.1635 (0.0037)	0.0791 (0.0052)	0.9270 (0.0082)	0.0330 (0.0015)
α_1	0.9621	0.2344	0.2346 (0.0052)	-0.0379 (0.0074)	0.9440 (0.0073)	0.0564 (0.0027)
α_2	0.1972	0.0093	0.0098 (0.0002)	-0.0028 (0.0003)	0.9280 (0.0082)	0.0001 (0.0000)
**Model D: ME (No adjustment)**						
α_0	0.0690	0.1686	0.1621 (0.0036)	0.0690 (0.0051)	0.9300 (0.0081)	0.0310 (0.0014)
α_1	0.9669	0.2350	0.2343 (0.0052)	-0.0331 (0.0074)	0.9430 (0.0073)	0.0560 (0.0027)
α_2	0.2018	0.0065	0.0065 (0.0001)	0.0018 (0.0002)	0.9320 (0.0080)	0.0000 (0.0000)
**Model E: GEE (IIVW)**						
α_0	1.1903	0.2969	0.2966 (0.0066)	1.1903 (0.0094)	0.0020 (0.0014)	1.5047 (0.0237)
α_1	1.0446	0.4230	0.4420 (0.0099)	0.0446 (0.0140)	0.9340 (0.0079)	0.1972 (0.0100)
α_2	0.2343	0.0247	0.0272 (0.0006)	0.0343 (0.0009)	0.6380 (0.0152)	0.0019 (0.0001)

Table B.10: Results for DGM: JM ($\gamma = 3.00, \lambda = 0.05$) with regular visits

	Estimate	SE	Empirical SE	Bias	Coverage	MSE
**Model A: JM**						
α_0	-0.0103	0.3118	0.3048 (0.0068)	-0.0103 (0.0096)	0.9570 (0.0064)	0.0929 (0.0039)
α_1	1.0190	0.4387	0.4367 (0.0098)	0.0190 (0.0138)	0.9540 (0.0066)	0.1909 (0.0085)
α_2	0.2000	0.0098	0.0099 (0.0002)	0.0000 (0.0003)	0.9429 (0.0073)	0.0001 (0.0000)
**Model B: ME (Total)**						
α_0	0.2867	0.2823	0.3078 (0.0069)	0.2867 (0.0097)	0.7960 (0.0127)	0.1768 (0.0070)
α_1	0.4269	0.4016	0.4059 (0.0091)	-0.5731 (0.0128)	0.6990 (0.0145)	0.4930 (0.0171)
α_2	0.1985	0.0098	0.0099 (0.0002)	-0.0015 (0.0003)	0.9500 (0.0069)	0.0001 (0.0000)
**Model C: ME (Cumulative)**						
α_0	-0.0271	0.3109	0.3052 (0.0068)	-0.0271 (0.0097)	0.9530 (0.0067)	0.0938 (0.0040)
α_1	1.0049	0.4370	0.4359 (0.0098)	0.0049 (0.0138)	0.9510 (0.0068)	0.1899 (0.0084)
α_2	0.1669	0.0358	0.0371 (0.0008)	-0.0331 (0.0012)	0.8480 (0.0114)	0.0025 (0.0001)
**Model D: ME (No adjustment)**						
α_0	-0.0104	0.3118	0.3046 (0.0068)	-0.0104 (0.0096)	0.9570 (0.0064)	0.0928 (0.0039)
α_1	1.0186	0.4387	0.4367 (0.0098)	0.0186 (0.0138)	0.9540 (0.0066)	0.1908 (0.0085)
α_2	0.2001	0.0098	0.0099 (0.0002)	0.0001 (0.0003)	0.9410 (0.0075)	0.0001 (0.0000)
**Model E: GEE (IIVW)**						
α_0	0.1576	0.3473	0.3467 (0.0078)	0.1576 (0.0110)	0.9360 (0.0077)	0.1449 (0.0063)
α_1	1.3071	0.5131	0.5227 (0.0117)	0.3071 (0.0165)	0.9110 (0.0090)	0.3672 (0.0164)
α_2	0.2157	0.0316	0.0324 (0.0007)	0.0157 (0.0010)	0.9170 (0.0087)	0.0013 (0.0001)

Bias, coverage, and mean squared error of the variance of the random effects u and v and the residual variance of ϵ are depicted in Figures B.3, B.4, B.5 and in Tables B.11 to B.20.

Table B.11: Results for DGM: Γ distribution not depending on treatment

	Estimate	SE	Empirical SE	Bias	Coverage	MSE
**Model A: JM**						
2	NA	NA	NA (0.0013)	NA (0.0018)	NA (0.0000)	NA (0.0028)
3	0.4815	0.0744	0.0756 (0.0017)	-0.0185 (0.0024)	0.9230 (0.0084)	0.0061 (0.0003)
V(ϵ)	0.9980	0.0519	0.0510 (0.0011)	-0.0020 (0.0016)	0.9490 (0.0070)	0.0026 (0.0001)
**Model B: ME (Total)**						
2	NA	NA	NA (NaN)	NA (NaN)	NA (NaN)	NA (NaN)
3	0.4866	0.0736	0.0747 (0.0017)	-0.0134 (0.0024)	0.9280 (0.0082)	0.0058 (0.0003)
V(ϵ)	0.9980	0.0519	0.0510 (0.0011)	-0.0020 (0.0016)	0.9500 (0.0069)	0.0026 (0.0001)
**Model C: ME (Cumulative)**						
2	NA	NA	NA (NaN)	NA (NaN)	NA (NaN)	NA (NaN)
3	0.4895	0.0740	0.0748 (0.0017)	-0.0105 (0.0024)	0.9340 (0.0079)	0.0057 (0.0003)
V(ϵ)	0.9971	0.0519	0.0511 (0.0011)	-0.0029 (0.0016)	0.9500 (0.0069)	0.0026 (0.0001)
**Model D: ME (No adjustment)**						
2	NA	NA	NA (NaN)	NA (NaN)	NA (NaN)	NA (NaN)
3	0.4905	0.0740	0.0747 (0.0017)	-0.0095 (0.0024)	0.9330 (0.0079)	0.0057 (0.0002)
V(ϵ)	0.9980	0.0519	0.0510 (0.0011)	-0.0020 (0.0016)	0.9510 (0.0068)	0.0026 (0.0001)
**Model E: GEE (IIVW)**						
2	—	—	—	—	—	—
3	—	—	—	—	—	—
V(ϵ)	—	—	—	—	—	—

Table B.12: Results for DGM: JM ($\gamma = 0.00, \lambda = 0.10$)

	Estimate	SE	Empirical SE	Bias	Coverage	MSE
**Model A: JM**						
2	NA	NA	NA (0.0067)	NA (0.0095)	NA (0.0124)	NA (0.0084)
3	0.4868	0.0912	0.0896 (0.0020)	-0.0132 (0.0028)	0.9440 (0.0073)	0.0082 (0.0003)
V(ϵ)	0.9943	0.0640	0.0654 (0.0015)	-0.0057 (0.0021)	0.9360 (0.0077)	0.0043 (0.0002)
**Model B: ME (Total)**						
2	NA	NA	NA (NaN)	NA (NaN)	NA (NaN)	NA (NaN)
3	0.4877	0.0912	0.0899 (0.0020)	-0.0123 (0.0028)	0.9440 (0.0073)	0.0082 (0.0003)
V(ϵ)	0.9951	0.0641	0.0655 (0.0015)	-0.0049 (0.0021)	0.9340 (0.0079)	0.0043 (0.0002)
**Model C: ME (Cumulative)**						
2	NA	NA	NA (NaN)	NA (NaN)	NA (NaN)	NA (NaN)
3	0.4934	0.0918	0.0902 (0.0020)	-0.0066 (0.0029)	0.9550 (0.0066)	0.0082 (0.0003)
V(ϵ)	0.9931	0.0640	0.0655 (0.0015)	-0.0069 (0.0021)	0.9320 (0.0080)	0.0043 (0.0002)
**Model D: ME (No adjustment)**						
2	NA	NA	NA (NaN)	NA (NaN)	NA (NaN)	NA (NaN)
3	0.4943	0.0916	0.0898 (0.0020)	-0.0057 (0.0028)	0.9530 (0.0067)	0.0081 (0.0003)
V(ϵ)	0.9944	0.0640	0.0653 (0.0015)	-0.0056 (0.0021)	0.9350 (0.0078)	0.0043 (0.0002)
**Model E: GEE (IIVW)**						
2	—	—	—	—	—	—
3	—	—	—	—	—	—
V(ϵ)	—	—	—	—	—	—

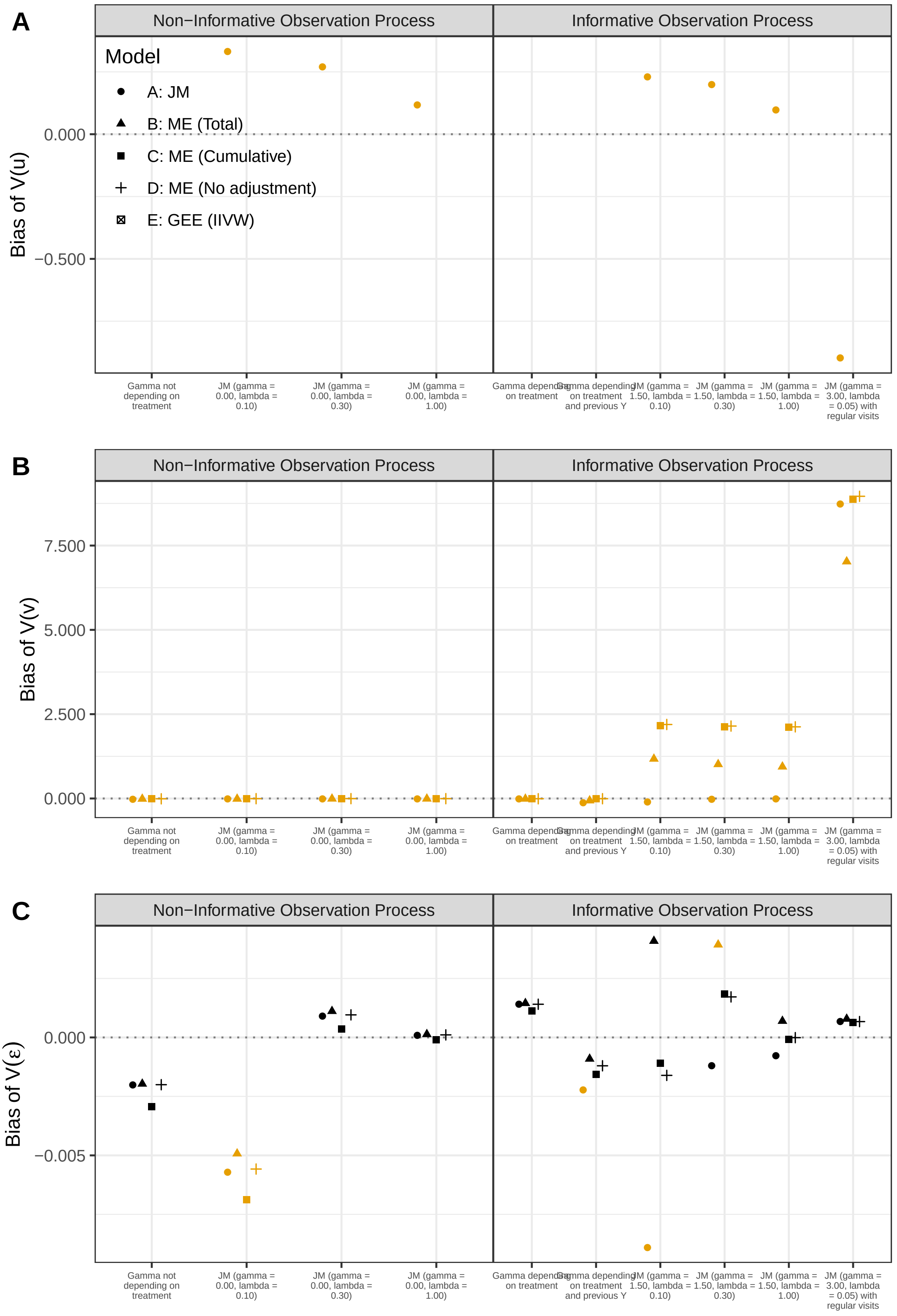

Figure B.3: Bias of variance terms for the random effects u, v and the residual error term ϵ. Orange colour identifies scenarios where bias was statistically significantly different than 0, using Z-tests and estimated Monte Carlo standard errors.

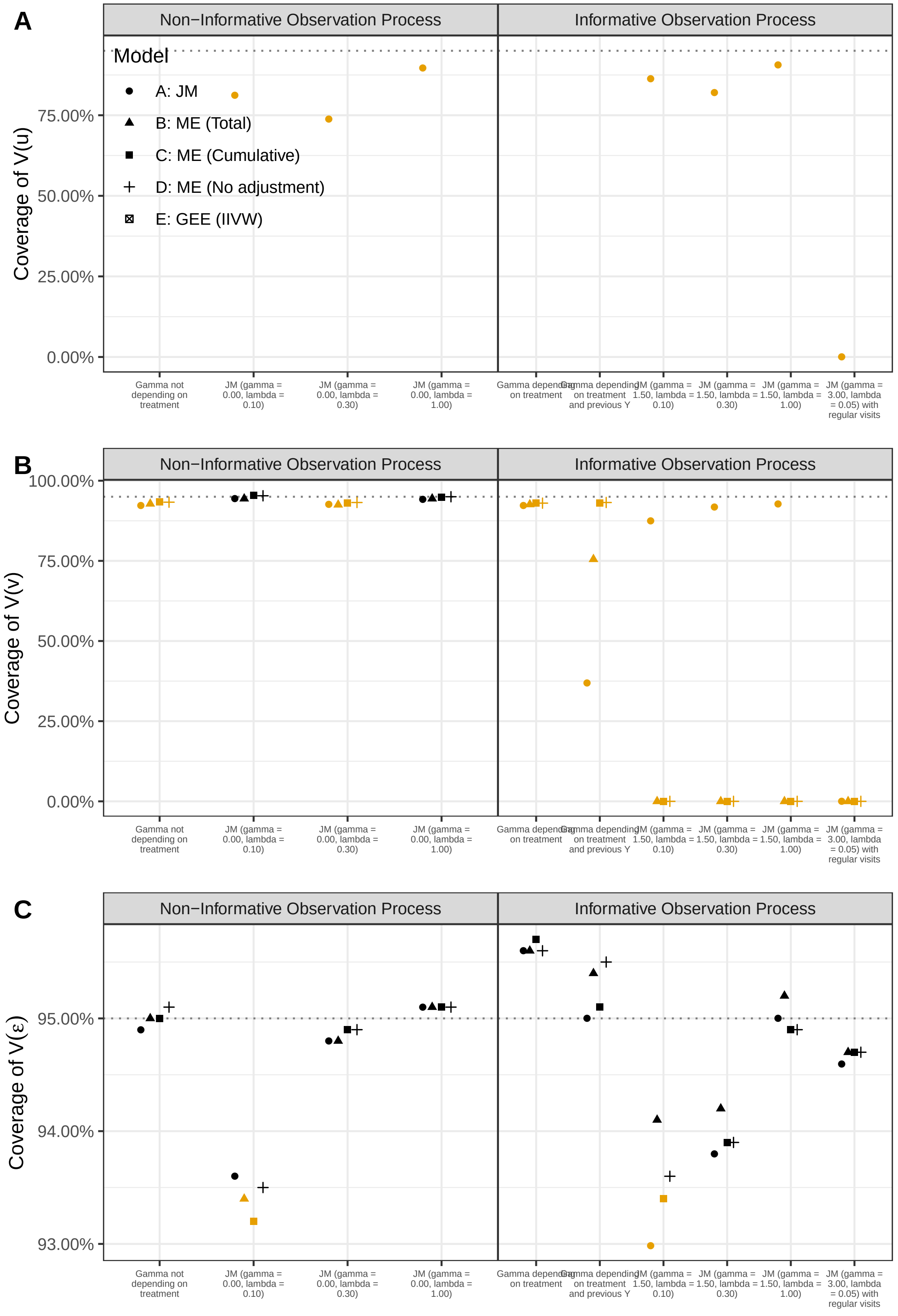

Figure B.4: Coverage probability of variance terms for the random effects u, v and the residual error term ϵ. Orange colour identifies scenarios where coverage probability was statistically significantly different than 95%, using Z-tests and estimated Monte Carlo standard errors.

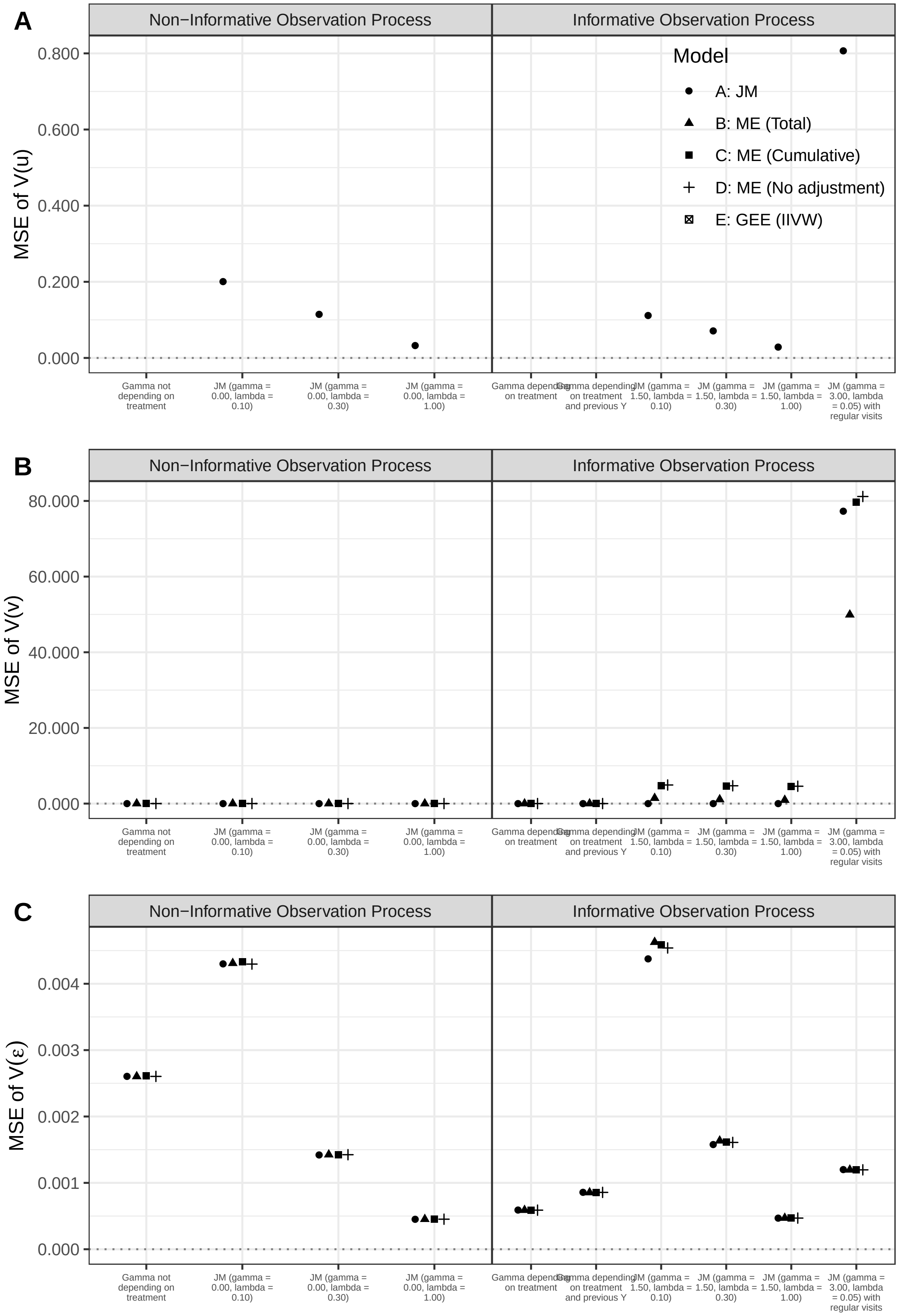

Figure B.5: Mean squared error of variance terms for the random effects u, v and the residual error term ϵ.

Table B.13: Results for DGM: JM ($\gamma = 0.00, \lambda = 0.30$)

	Estimate	SE	Empirical SE	Bias	Coverage	MSE
**Model A: JM**						
2	NA	NA	NA (0.0046)	NA (0.0065)	NA (0.0139)	NA (0.0045)
3	0.4913	0.0713	0.0737 (0.0016)	-0.0087 (0.0023)	0.9260 (0.0083)	0.0055 (0.0002)
V(ϵ)	1.0009	0.0382	0.0377 (0.0008)	0.0009 (0.0012)	0.9480 (0.0070)	0.0014 (0.0001)
**Model B: ME (Total)**						
2	NA	NA	NA (NaN)	NA (NaN)	NA (NaN)	NA (NaN)
3	0.4912	0.0713	0.0737 (0.0016)	-0.0088 (0.0023)	0.9250 (0.0083)	0.0055 (0.0003)
V(ϵ)	1.0011	0.0382	0.0378 (0.0008)	0.0011 (0.0012)	0.9480 (0.0070)	0.0014 (0.0001)
**Model C: ME (Cumulative)**						
2	NA	NA	NA (NaN)	NA (NaN)	NA (NaN)	NA (NaN)
3	0.4949	0.0717	0.0739 (0.0017)	-0.0051 (0.0023)	0.9310 (0.0080)	0.0055 (0.0003)
V(ϵ)	1.0004	0.0382	0.0378 (0.0008)	0.0004 (0.0012)	0.9490 (0.0070)	0.0014 (0.0001)
**Model D: ME (No adjustment)**						
2	NA	NA	NA (NaN)	NA (NaN)	NA (NaN)	NA (NaN)
3	0.4954	0.0717	0.0737 (0.0016)	-0.0046 (0.0023)	0.9320 (0.0080)	0.0054 (0.0002)
V(ϵ)	1.0010	0.0382	0.0377 (0.0008)	0.0010 (0.0012)	0.9490 (0.0070)	0.0014 (0.0001)
**Model E: GEE (IIVW)**						
2	—	—	—	—	—	—
3	—	—	—	—	—	—
V(ϵ)	—	—	—	—	—	—

Table B.14: Results for DGM: JM ($\gamma = 0.00, \lambda = 1.00$)

	Estimate	SE	Empirical SE	Bias	Coverage	MSE
**Model A: JM**						
2	NA	NA	NA (0.0031)	NA (0.0044)	NA (0.0096)	NA (0.0013)
3	0.4914	0.0591	0.0579 (0.0013)	-0.0086 (0.0018)	0.9420 (0.0074)	0.0034 (0.0001)
V(ϵ)	1.0001	0.0216	0.0213 (0.0005)	0.0001 (0.0007)	0.9510 (0.0068)	0.0005 (0.0000)
**Model B: ME (Total)**						
2	NA	NA	NA (NaN)	NA (NaN)	NA (NaN)	NA (NaN)
3	0.4912	0.0591	0.0579 (0.0013)	-0.0088 (0.0018)	0.9440 (0.0073)	0.0034 (0.0001)
V(ϵ)	1.0001	0.0216	0.0213 (0.0005)	0.0001 (0.0007)	0.9510 (0.0068)	0.0005 (0.0000)
**Model C: ME (Cumulative)**						
2	NA	NA	NA (NaN)	NA (NaN)	NA (NaN)	NA (NaN)
3	0.4944	0.0594	0.0583 (0.0013)	-0.0056 (0.0018)	0.9490 (0.0070)	0.0034 (0.0001)
V(ϵ)	0.9999	0.0216	0.0213 (0.0005)	-0.0001 (0.0007)	0.9510 (0.0068)	0.0005 (0.0000)
**Model D: ME (No adjustment)**						
2	NA	NA	NA (NaN)	NA (NaN)	NA (NaN)	NA (NaN)
3	0.4946	0.0594	0.0583 (0.0013)	-0.0054 (0.0018)	0.9500 (0.0069)	0.0034 (0.0001)
V(ϵ)	1.0001	0.0216	0.0213 (0.0005)	0.0001 (0.0007)	0.9510 (0.0068)	0.0005 (0.0000)
**Model E: GEE (IIVW)**						
2	—	—	—	—	—	—
3	—	—	—	—	—	—
V(ϵ)	—	—	—	—	—	—

Table B.15: Results for DGM: Γ distribution depending on treatment

	Estimate	SE	Empirical SE	Bias	Coverage	MSE
**Model A: JM**						
2	NA	NA	NA (0.0008)	NA (0.0012)	NA (0.0000)	NA (0.0018)
3	0.4877	0.0608	0.0624 (0.0014)	-0.0123 (0.0020)	0.9230 (0.0084)	0.0040 (0.0002)
V(ϵ)	1.0014	0.0249	0.0242 (0.0005)	0.0014 (0.0008)	0.9560 (0.0065)	0.0006 (0.0000)
**Model B: ME (Total)**						
2	NA	NA	NA (NaN)	NA (NaN)	NA (NaN)	NA (NaN)
3	0.4885	0.0608	0.0626 (0.0014)	-0.0115 (0.0020)	0.9260 (0.0083)	0.0040 (0.0002)
V(ϵ)	1.0014	0.0249	0.0242 (0.0005)	0.0014 (0.0008)	0.9560 (0.0065)	0.0006 (0.0000)
**Model C: ME (Cumulative)**						
2	NA	NA	NA (NaN)	NA (NaN)	NA (NaN)	NA (NaN)
3	0.4920	0.0611	0.0627 (0.0014)	-0.0080 (0.0020)	0.9310 (0.0080)	0.0040 (0.0002)
V(ϵ)	1.0011	0.0249	0.0242 (0.0005)	0.0011 (0.0008)	0.9570 (0.0064)	0.0006 (0.0000)
**Model D: ME (No adjustment)**						
2	NA	NA	NA (NaN)	NA (NaN)	NA (NaN)	NA (NaN)
3	0.4922	0.0611	0.0626 (0.0014)	-0.0078 (0.0020)	0.9300 (0.0081)	0.0040 (0.0002)
V(ϵ)	1.0014	0.0249	0.0242 (0.0005)	0.0014 (0.0008)	0.9560 (0.0065)	0.0006 (0.0000)
**Model E: GEE (IIVW)**						
2	—	—	—	—	—	—
3	—	—	—	—	—	—
V(ϵ)	—	—	—	—	—	—

Table B.16: Results for DGM: Γ distribution depending on treatment and previous Y

	Estimate	SE	Empirical SE	Bias	Coverage	MSE
**Model A: JM**						
2	NA	NA	NA (0.0009)	NA (0.0013)	NA (0.0000)	NA (0.0019)
3	0.3733	0.0545	0.0530 (0.0012)	-0.1267 (0.0017)	0.3690 (0.0153)	0.0189 (0.0004)
V(ϵ)	0.9978	0.0290	0.0292 (0.0007)	-0.0022 (0.0009)	0.9500 (0.0069)	0.0009 (0.0000)
**Model B: ME (Total)**						
2	NA	NA	NA (NaN)	NA (NaN)	NA (NaN)	NA (NaN)
3	0.4377	0.0574	0.0567 (0.0013)	-0.0623 (0.0018)	0.7550 (0.0136)	0.0071 (0.0002)
V(ϵ)	0.9991	0.0291	0.0292 (0.0007)	-0.0009 (0.0009)	0.9540 (0.0066)	0.0009 (0.0000)
**Model C: ME (Cumulative)**						
2	NA	NA	NA (NaN)	NA (NaN)	NA (NaN)	NA (NaN)
3	0.4928	0.0635	0.0641 (0.0014)	-0.0072 (0.0020)	0.9310 (0.0080)	0.0042 (0.0002)
V(ϵ)	0.9984	0.0291	0.0292 (0.0007)	-0.0016 (0.0009)	0.9510 (0.0068)	0.0009 (0.0000)
**Model D: ME (No adjustment)**						
2	NA	NA	NA (NaN)	NA (NaN)	NA (NaN)	NA (NaN)
3	0.4931	0.0630	0.0639 (0.0014)	-0.0069 (0.0020)	0.9320 (0.0080)	0.0041 (0.0002)
V(ϵ)	0.9988	0.0291	0.0293 (0.0007)	-0.0012 (0.0009)	0.9550 (0.0066)	0.0009 (0.0000)
**Model E: GEE (IIVW)**						
2	—	—	—	—	—	—
3	—	—	—	—	—	—
V(ϵ)	—	—	—	—	—	—

Table B.17: Results for DGM: JM ($\gamma = 1.50, \lambda = 0.10$)

	Estimate	SE	Empirical SE	Bias	Coverage	MSE
**Model A: JM**						
2	NA	NA	NA (0.0056)	NA (0.0079)	NA (0.0111)	NA (0.0052)
3	0.4007	0.1654	0.1617 (0.0037)	-0.0993 (0.0052)	0.8743 (0.0107)	0.0360 (0.0014)
V(ϵ)	0.9911	0.0641	0.0656 (0.0015)	-0.0089 (0.0021)	0.9298 (0.0083)	0.0044 (0.0002)
**Model B: ME (Total)**						
2	NA	NA	NA (NaN)	NA (NaN)	NA (NaN)	NA (NaN)
3	1.6740	0.2223	0.2558 (0.0057)	1.1740 (0.0081)	0.0000 (0.0000)	1.4436 (0.0203)
V(ϵ)	1.0041	0.0660	0.0679 (0.0015)	0.0041 (0.0021)	0.9410 (0.0075)	0.0046 (0.0002)
**Model C: ME (Cumulative)**						
2	NA	NA	NA (NaN)	NA (NaN)	NA (NaN)	NA (NaN)
3	2.6609	0.3222	0.3238 (0.0072)	2.1609 (0.0102)	0.0000 (0.0000)	4.7741 (0.0454)
V(ϵ)	0.9989	0.0657	0.0678 (0.0015)	-0.0011 (0.0021)	0.9340 (0.0079)	0.0046 (0.0002)
**Model D: ME (No adjustment)**						
2	NA	NA	NA (NaN)	NA (NaN)	NA (NaN)	NA (NaN)
3	2.6941	0.3227	0.3173 (0.0071)	2.1941 (0.0100)	0.0000 (0.0000)	4.9148 (0.0451)
V(ϵ)	0.9984	0.0654	0.0674 (0.0015)	-0.0016 (0.0021)	0.9360 (0.0077)	0.0045 (0.0002)
**Model E: GEE (IIVW)**						
2	—	—	—	—	—	—
3	—	—	—	—	—	—
V(ϵ)	—	—	—	—	—	—

Table B.18: Results for DGM: JM ($\gamma = 1.50, \lambda = 0.30$)

	Estimate	SE	Empirical SE	Bias	Coverage	MSE
**Model A: JM**						
2	NA	NA	NA (0.0040)	NA (0.0056)	NA (0.0121)	NA (0.0029)
3	0.4747	0.1045	0.1076 (0.0024)	-0.0253 (0.0034)	0.9180 (0.0087)	0.0122 (0.0006)
V(ϵ)	0.9988	0.0384	0.0397 (0.0009)	-0.0012 (0.0013)	0.9380 (0.0076)	0.0016 (0.0001)
**Model B: ME (Total)**						
2	NA	NA	NA (NaN)	NA (NaN)	NA (NaN)	NA (NaN)
3	1.5130	0.1840	0.2553 (0.0057)	1.0130 (0.0081)	0.0000 (0.0000)	1.0912 (0.0181)
V(ϵ)	1.0039	0.0388	0.0403 (0.0009)	0.0039 (0.0013)	0.9420 (0.0074)	0.0016 (0.0001)
**Model C: ME (Cumulative)**						
2	NA	NA	NA (NaN)	NA (NaN)	NA (NaN)	NA (NaN)
3	2.6216	0.2969	0.3148 (0.0070)	2.1216 (0.0100)	0.0000 (0.0000)	4.6000 (0.0433)
V(ϵ)	1.0018	0.0387	0.0402 (0.0009)	0.0018 (0.0013)	0.9390 (0.0076)	0.0016 (0.0001)
**Model D: ME (No adjustment)**						
2	NA	NA	NA (NaN)	NA (NaN)	NA (NaN)	NA (NaN)
3	2.6477	0.2977	0.3088 (0.0069)	2.1477 (0.0098)	0.0000 (0.0000)	4.7079 (0.0430)
V(ϵ)	1.0017	0.0387	0.0401 (0.0009)	0.0017 (0.0013)	0.9390 (0.0076)	0.0016 (0.0001)
**Model E: GEE (IIVW)**						
2	—	—	—	—	—	—
3	—	—	—	—	—	—
V(ϵ)	—	—	—	—	—	—

Table B.19: Results for DGM: JM ($\gamma = 1.50, \lambda = 1.00$)

	Estimate	SE	Empirical SE	Bias	Coverage	MSE
**Model A: JM**						
2	NA	NA	NA (0.0031)	NA (0.0043)	NA (0.0092)	NA (0.0013)
3	0.4914	0.0741	0.0740 (0.0017)	-0.0086 (0.0023)	0.9280 (0.0082)	0.0055 (0.0002)
V(ϵ)	0.9992	0.0216	0.0217 (0.0005)	-0.0008 (0.0007)	0.9500 (0.0069)	0.0005 (0.0000)
**Model B: ME (Total)**						
2	NA	NA	NA (NaN)	NA (NaN)	NA (NaN)	NA (NaN)
3	1.4420	0.1611	0.2330 (0.0052)	0.9420 (0.0074)	0.0000 (0.0000)	0.9416 (0.0151)
V(ϵ)	1.0007	0.0217	0.0217 (0.0005)	0.0007 (0.0007)	0.9520 (0.0068)	0.0005 (0.0000)
**Model C: ME (Cumulative)**						
2	NA	NA	NA (NaN)	NA (NaN)	NA (NaN)	NA (NaN)
3	2.6087	0.2786	0.2779 (0.0062)	2.1087 (0.0088)	0.0000 (0.0000)	4.5239 (0.0374)
V(ϵ)	0.9999	0.0216	0.0217 (0.0005)	-0.0001 (0.0007)	0.9490 (0.0070)	0.0005 (0.0000)
**Model D: ME (No adjustment)**						
2	NA	NA	NA (NaN)	NA (NaN)	NA (NaN)	NA (NaN)
3	2.6243	0.2792	0.2749 (0.0062)	2.1243 (0.0087)	0.0000 (0.0000)	4.5883 (0.0373)
V(ϵ)	1.0000	0.0216	0.0217 (0.0005)	-0.0000 (0.0007)	0.9490 (0.0070)	0.0005 (0.0000)
**Model E: GEE (IIVW)**						
2	—	—	—	—	—	—
3	—	—	—	—	—	—
V(ϵ)	—	—	—	—	—	—

Table B.20: Results for DGM: JM ($\gamma = 3.00, \lambda = 0.05$) with regular visits

	Estimate	SE	Empirical SE	Bias	Coverage	MSE
**Model A: JM**						
2	NA	NA	NA (0.0007)	NA (0.0009)	NA (0.0000)	NA (0.0016)
3	9.2358	0.9716	1.0114 (0.0226)	8.7358 (0.0320)	0.0000 (0.0000)	77.3352 (0.5583)
V(ϵ)	1.0007	0.0349	0.0346 (0.0008)	0.0007 (0.0011)	0.9459 (0.0072)	0.0012 (0.0001)
**Model B: ME (Total)**						
2	NA	NA	NA (NaN)	NA (NaN)	NA (NaN)	NA (NaN)
3	7.5247	0.7680	0.7599 (0.0170)	7.0247 (0.0240)	0.0000 (0.0000)	49.9237 (0.3440)
V(ϵ)	1.0008	0.0350	0.0346 (0.0008)	0.0008 (0.0011)	0.9470 (0.0071)	0.0012 (0.0001)
**Model C: ME (Cumulative)**						
2	NA	NA	NA (NaN)	NA (NaN)	NA (NaN)	NA (NaN)
3	9.3746	0.9573	0.9426 (0.0211)	8.8746 (0.0298)	0.0000 (0.0000)	79.6456 (0.5335)
V(ϵ)	1.0006	0.0350	0.0346 (0.0008)	0.0006 (0.0011)	0.9470 (0.0071)	0.0012 (0.0001)
**Model D: ME (No adjustment)**						
2	NA	NA	NA (NaN)	NA (NaN)	NA (NaN)	NA (NaN)
3	9.4606	0.9616	0.9394 (0.0210)	8.9606 (0.0297)	0.0000 (0.0000)	81.1735 (0.5363)
V(ϵ)	1.0007	0.0349	0.0346 (0.0008)	0.0007 (0.0011)	0.9470 (0.0071)	0.0012 (0.0001)
**Model E: GEE (IIVW)**						
2	—	—	—	—	—	—
3	—	—	—	—	—	—
V(ϵ)	—	—	—	—	—	—

Results for the association parameter γ are tabulated here in Table B.21 and depicted in Figure B.6. Values tabulated in red are summary statistics that are statistically significantly different than their target value (0 for bias, 95% for coverage), using Z-tests and estimated Monte Carlo standard errors.

Table B.21: Results for the association parameter γ

True γ	True λ	Estimate	SE	Empirical SE	Bias	Coverage	MSE
0.00	0.10	-0.0037	0.0785	0.0810 (0.0018)	-0.0037 (0.0026)	0.9450 (0.0072)	0.0066 (0.0003)
0.00	0.30	0.0007	0.0635	0.0617 (0.0014)	0.0007 (0.0019)	0.9530 (0.0067)	0.0038 (0.0002)
0.00	1.00	0.0015	0.0571	0.0576 (0.0013)	0.0015 (0.0018)	0.9510 (0.0068)	0.0033 (0.0001)
1.50	0.10	1.4089	0.1419	0.1439 (0.0033)	-0.0911 (0.0047)	0.8346 (0.0120)	0.0290 (0.0011)
1.50	0.30	1.3917	0.0917	0.0920 (0.0021)	-0.1083 (0.0029)	0.7460 (0.0138)	0.0202 (0.0007)
1.50	1.00	1.4334	0.0696	0.0724 (0.0016)	-0.0666 (0.0023)	0.8080 (0.0125)	0.0097 (0.0004)
3.00	0.05	-0.7289	1.1352	1.4334 (0.0321)	-3.7289 (0.0454)	0.1231 (0.0104)	15.9574 (0.3742)

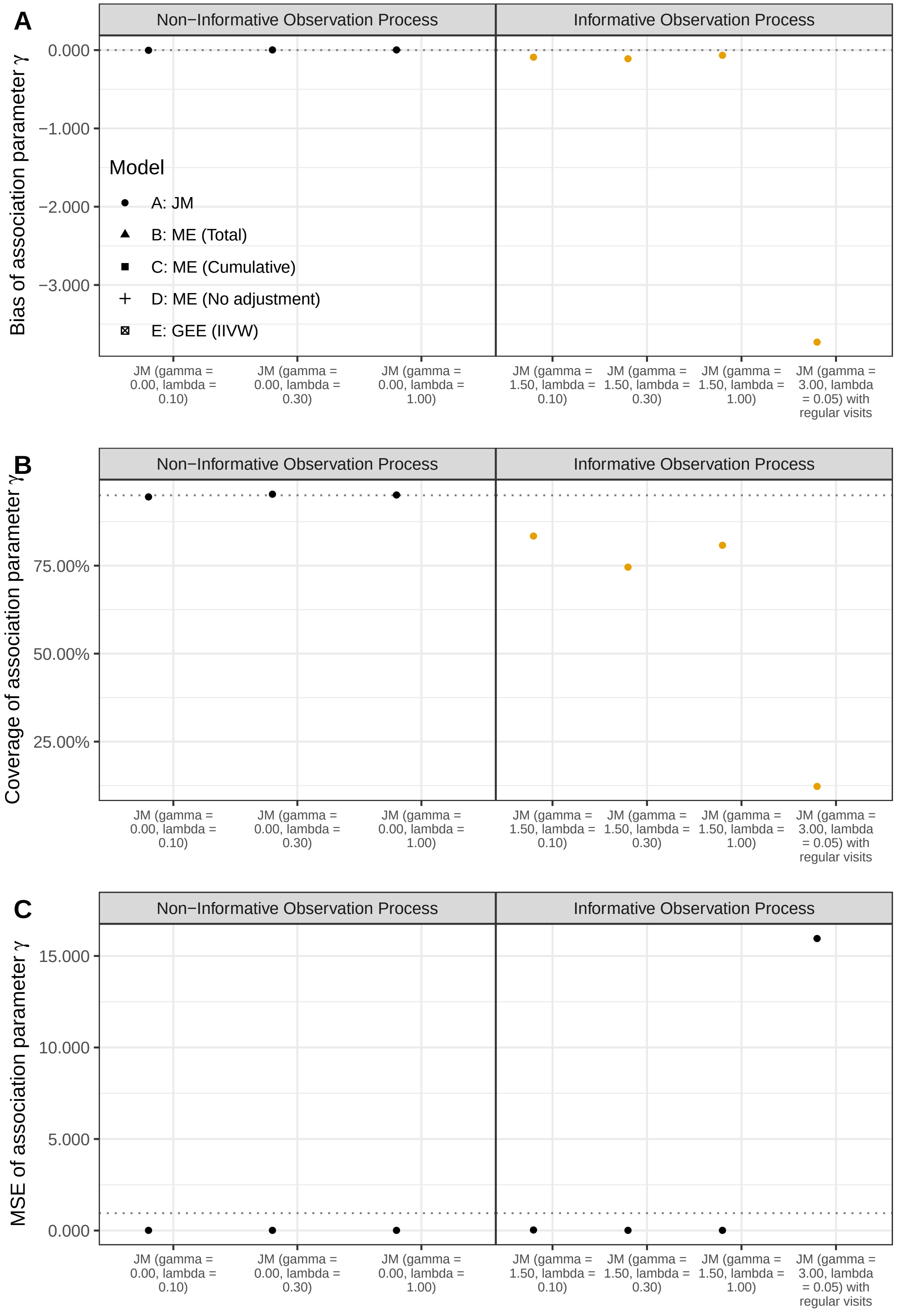

Figure B.6: Bias (A), coverage probability (B), and mean squared error (C) of the association parameter γ. Orange colour identifies scenarios where the summary statistic was statistically significantly different than the target value (0 for bias, 95% for coverage), using Z-tests and estimated Monte Carlo standard errors.

Finally, convergence rates for each model under each data-generating mechanism are presented in Table B.22.

Table B.22: Number and percentage of models converging under each data-generating mechanism. Model A is the joint model, model B is the mixed model adjusting for the total number of measurements, model C is the mixed model adjusting for the cumulative number of measurements, model D is the mixed model with no further adjustment, and model E is the marginal model fitted using GEE and IIVW.

Data-Generating Mechanism	Model A	Model B	Model C	Model D	Model E
JM ($\gamma = 0.00, \lambda = 0.10$)	1,000 (100%)	1,000 (100%)	1,000 (100%)	1,000 (100%)	1,000 (100%)
JM ($\gamma = 0.00, \lambda = 0.30$)	1,000 (100%)	1,000 (100%)	1,000 (100%)	1,000 (100%)	1,000 (100%)
JM ($\gamma = 0.00, \lambda = 1.00$)	1,000 (100%)	1,000 (100%)	1,000 (100%)	1,000 (100%)	1,000 (100%)
JM ($\gamma = 1.50, \lambda = 0.10$)	955 (96%)	1,000 (100%)	1,000 (100%)	1,000 (100%)	1,000 (100%)
JM ($\gamma = 1.50, \lambda = 0.30$)	1,000 (100%)	1,000 (100%)	1,000 (100%)	1,000 (100%)	1,000 (100%)
JM ($\gamma = 1.50, \lambda = 1.00$)	1,000 (100%)	1,000 (100%)	1,000 (100%)	1,000 (100%)	1,000 (100%)
Γ distribution not depending on treatment	1,000 (100%)	1,000 (100%)	1,000 (100%)	1,000 (100%)	1,000 (100%)
Γ distribution depending on treatment	1,000 (100%)	1,000 (100%)	1,000 (100%)	1,000 (100%)	1,000 (100%)
Γ distribution depending on treatment and previous Y	1,000 (100%)	1,000 (100%)	1,000 (100%)	1,000 (100%)	1,000 (100%)
JM ($\gamma = 3.00, \lambda = 0.05$) with regular visits	999 (100%)	1,000 (100%)	1,000 (100%)	1,000 (100%)	1,000 (100%)
